# Review of State Estimation Methods for Autonomous Ground Vehicles: Perspectives on Estimation Objects, Vehicle Characteristics, and Key Algorithms

**DOI:** 10.3390/s25133927

**Published:** 2025-06-24

**Authors:** Xiaoyu Wang, Te Chen, Renzhong Wang, Jiankang Lu, Guowei Dou

**Affiliations:** 1 School of Mechanical and Electrical Engineering, Suzhou Vocational University, Suzhou 215000, China; 91811@jssvc.edu.cn (R.W.); 91736@jssvc.edu.cn (J.L.); 2 Robotics and Intelligent Equipment Engineering Research Center of Jiangsu Province, Suzhou Vocational University, Suzhou 215000, China; 3 Automotive Engineering Research Institute, Jiangsu University, Zhenjiang 212013, China; 1000005564@ujs.edu.cn; 4 School of Automotive and Transportation Engineering, Jiangsu University, Zhenjiang 212013, China; dgwujs@126.com

**Keywords:** vehicle observer, state estimation, vehicle characteristics, data driven, filtering algorithm

## Abstract

This paper reviews research on vehicle driving state estimation research. Based on the discussion of the importance, development history, and application fields of this topic of research, it focuses on analyzing vehicle state estimation techniques from different perspectives, namely (1) from the perspective of the estimation objects, including vehicle attitude and driving state estimations, chassis component key dynamic parameter estimations, and vehicle driving environment state estimations; (2) from the perspective of vehicle characteristics, including vehicle dynamics coupling characteristics, vehicle multi-source information redundancy characteristics, and vehicle state transition characteristics; (3) from the perspective of key estimation algorithms, including model-based Kalman filtering algorithms, data-driven machine learning algorithms, and optimization estimation algorithms combining mechanism-based and data-driven approaches. This manuscript helps interested readers to comprehensively understand the research progress, technical features, and future trends of vehicle state estimation technology from the perspective of overall architecture and subdomains.

## 1. Introduction

Vehicle state estimation technology, as one of the core technologies in modern vehicle engineering, is receiving increasing attention from the industry. This technology integrates advanced sensors and algorithms, providing real-time vehicle status information. Accurate acquisition and processing of information is crucial for improving the safety [1], stability [2], energy efficiency [3], and intelligence [4] of vehicles.

State estimation technology can provide accurate vehicle state information for vehicle control systems. In complex road environments, it is possible to rely on these real-time data to make appropriate control decisions, which will help achieve the higher-level autonomous driving functions for vehicles [5,6,7]. Meanwhile, accurate and reliable vehicle state estimation results can help to achieve efficient and safe operation of vehicles. Combined with the real-time status of the vehicle, the vehicle control system can achieve optimal steering control, braking control, suspension control, and dynamic drive distribution control based on the current vehicle status, thereby achieving the optimization of vehicle dynamics and active safety control functions [8,9]. Moreover, real-time monitoring of vehicle status and evaluation analysis can be achieved using state estimation methods, laying the foundation for vehicle fault-tolerant control. In addition, with the continuous development of technologies such as autonomous driving, big data, and artificial intelligence, vehicle state estimation technology will be combined with more advanced vehicle control technologies and applied to more driving scenarios, further improving the intelligence and motion control quality of vehicles.

The precise acquisition of vehicle status in early stages mainly relied on basic sensors and data processing technologies. Although this can provide the required vehicle status information to a certain extent, it is difficult to meet the growing demand for control applications due to its limitations in accuracy, real-time performance, and stability. With the continuous advancement of sensor technology, computing power, and data processing methods, vehicle state estimation technology is gradually developing and integrating more sensor information. The addition of many onboard sensors such as inertial measurement units, LiDAR, and cameras has made the methods of obtaining vehicle status data more diverse. At this point, model-based state observers are a common design approach [10,11,12]. Meanwhile, complex algorithms have been applied in data fusion and state estimation, including Kalman filtering, particle filtering, and recently emerging deep learning methods. The application of these algorithms significantly improves the accuracy and stability of vehicle state estimation.

At present, the vehicle state estimation technology is developing towards higher accuracy, stronger robustness, and lower cost. In the process of observer design, the coupling characteristics of vehicle dynamics, the estimation accuracy of the vehicle for complex extreme working conditions, and the dynamic adjustment ability for large-scale road conditions are the focus of current researchers [13,14]. With the development trend of vehicle electrification and intelligence, new high-precision sensors can offer richer and more accurate and reliable vehicle status information, providing more possibilities and optimization space for vehicle status estimation [15,16]. The introduction of data-driven optimization algorithms, especially the application of deep learning and reinforcement learning algorithms, provides a new direction for the development of vehicle state estimation technology. Data-driven technology and optimization algorithms can fit optimization models from the experimental training data, further improving the accuracy and efficiency of state estimation [17,18,19].

The vehicle state estimation technology has shown great potential in applications such as autonomous driving [20], active vehicle safety control [21], vehicle state monitoring, and fault-tolerant control [22]. However, current vehicle state estimation technology still faces some challenges and problems, such as high-precision and reliable vehicle dynamics modeling, robustness and adaptability issues of data fusion algorithms, and the adaptability issues of vehicle state estimation systems in different scenarios and complex environments, which have current technical difficulties that require further research and in-depth exploration [23,24,25]. In recent years, many scholars have made outstanding contributions in this field. With the continuous progress and innovation of technology in the future, we have reason to believe that vehicle state estimation technology will play a more important supporting role in the fields of intelligent driving and vehicle control.

**Figure 1:**
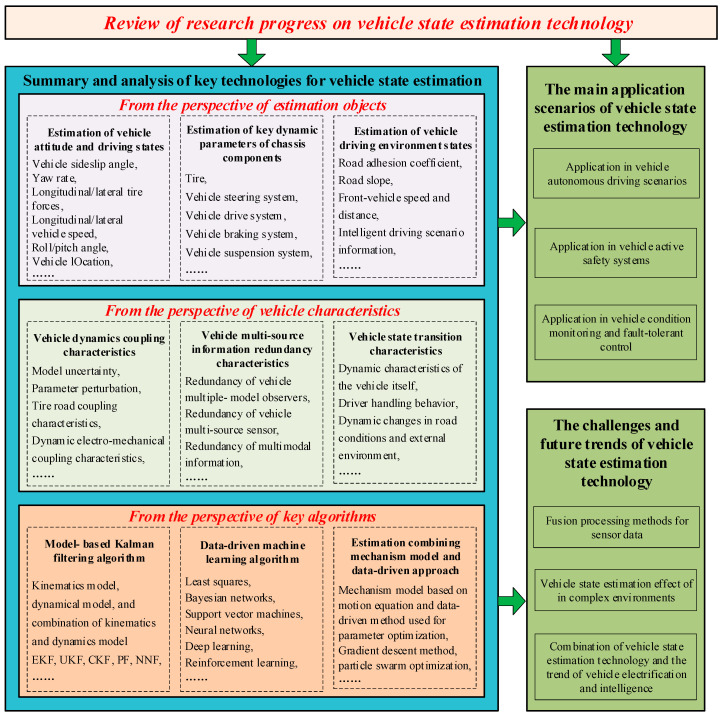
Main contents of this paper.

This paper focuses on the research progress of vehicle driving state estimation methods, and the main contents of this paper are shown in Figure 1. We review the state of the art for the latest vehicle state estimation technology and ideas from different perspectives. The rest of this paper is organized as follows. In Section 2, the research progress of vehicle state estimation from the perspective of estimation objects is analyzed. In Section 3, the research progress of vehicle state estimation from the perspective of vehicle characteristics is discussed. In Section 4, the research progress of vehicle state estimation from the perspective of key algorithms is summarized. In Section 5, the main application scenarios of vehicle state estimation technology are analyzed. In Section 6, the challenges and future trends of vehicle state estimation technology are discussed. Finally, conclusions are drawn in Section 7.

## 2. Vehicle State Estimation: From the Perspective of Estimation Objects

**Figure 2:**
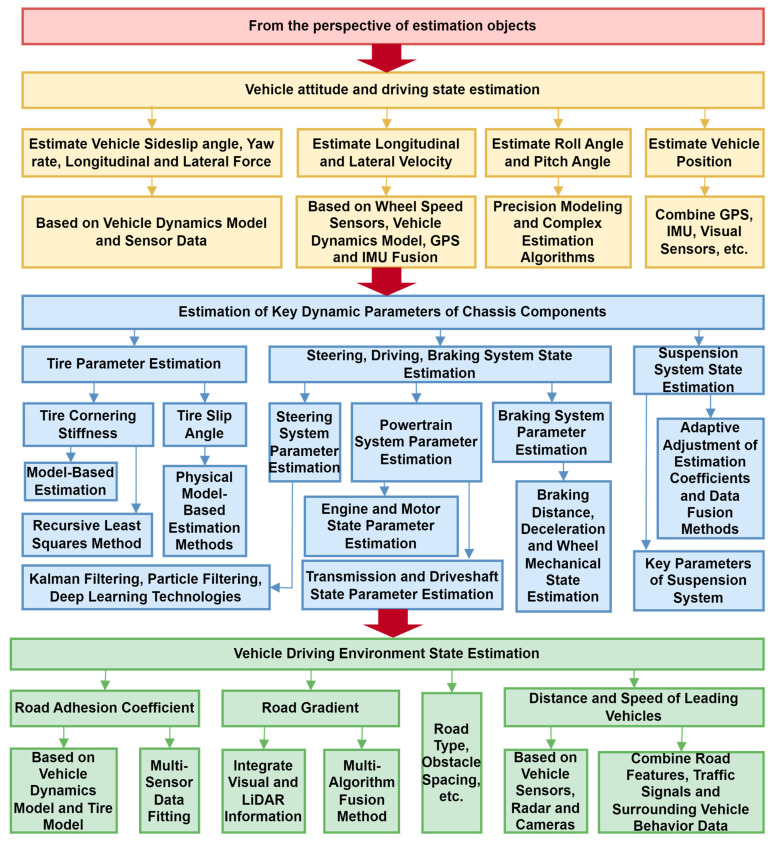
Vehicle state estimation: from the perspective of estimation objects.

Vehicle state estimation covers three core dimensions: attitude, chassis dynamic parameters, and driving environment, which are crucial for vehicle control and intelligence. The estimation of vehicle attitude and driving status is achieved through the fusion of multiple sensors (radar, GPS, IMU, etc.), which obtain key parameters, such as center of mass slip angle, yaw rate, longitudinal/lateral force, and velocity, in real time. The slip angle of the center of mass reflects the stability of the vehicle, which needs to be combined with the dynamic model and wheel speed and steering angle data. The yaw rate is indirectly calculated using a dynamic model, which directly affects stability control. Longitudinal/lateral velocities are estimated using wheel speed sensors or kinematic models, while position estimation relies on GPS and visual fusion to provide core positioning support for autonomous driving. Dynamic parameter estimation of chassis components focuses on tire, steering, braking, and suspension systems. The lateral stiffness and sideslip angle of the tire are optimized using model-driven methods such as recursive least squares and bilinear Kalman filtering to address the limitations of traditional empirical formulas. The steering system adopts Kalman filtering and deep learning to improve the accuracy of steering angle estimation. The braking system relies on wheel speed sensors and sliding mode algorithms to estimate braking pressure. The suspension system improves its parameter identification ability through adaptive data fusion. The estimation of driving environment state is based on factors such as road adhesion coefficient, slope, and obstacle spacing and is implemented by combining multiple sensors (LiDAR, camera) and dynamic models. The road adhesion coefficient is estimated using the slip ratio friction relationship or LuGre tire model. Slope estimation adopts visual LiDAR fusion or vehicle mass coupling model. The distance and speed of the vehicles ahead are detected through radar camera cooperation, and this information is combined with trajectory prediction algorithms to improve safety, such as Fan Pengfei’s road level estimation method based on energy consumption differences. These estimation techniques significantly improve the control accuracy and safety redundancy of vehicles under complex operating conditions using model data collaborative optimization, supporting the reliable operation of intelligent driving systems. The overall research content architecture of this chapter is shown in Figure 2.

### 2.1. Vehicle Attitude and Driving State Estimation

Vehicle attitude and driving state estimation is an important component of vehicle control, navigation, and active safety systems [26]. By utilizing various sensor data information, such as cameras, radars, GPS, etc., accurate estimation of key parameters such as center of mass sideslip angle, yaw rate, longitudinal force, lateral force, longitudinal speed, lateral speed, wheel angular velocity, and vehicle position can be achieved to comprehensively monitor and control the vehicle’s driving status, as shown in Table 1, thereby improving the safety, stability, and comfort of the vehicle [27].

The vehicle sideslip angle refers to the angle between the vehicle centroid velocity and the *X*-axis of the vehicle coordinate system [28]. It is an important parameter for describing vehicle stability and handling. The estimation of the center of mass sideslip angle is usually achieved by combining the vehicle dynamics model with sensor data (such as wheel speed, steering angle, etc.). The accuracy and real-time performance of estimation are crucial for vehicle stability control, path tracking, and active safety systems. The yaw rate describes the angular velocity of a vehicle rotating around its vertical axis, and its estimation accuracy is crucial for vehicle stability control [29]. It can be indirectly calculated using vehicle dynamics models and other sensor data. The estimation of longitudinal tire force is usually based on the vehicle dynamics model, combined with data from acceleration sensors, wheel speed sensors, etc. for calculation [30]. Accurate longitudinal tire force estimation is of great significance for vehicle control and energy management. The estimation of lateral tire force usually relies on data from vehicle dynamics models, wheel speed sensors, and lateral acceleration sensors, and accurate lateral force estimation is crucial for vehicle stability control, path tracking, and more [31].

The estimation of longitudinal vehicle speed can be achieved using various methods, including direct measurement based on wheel speed sensors, indirect calculation based on vehicle dynamics models, and fusion estimation based on GPS and inertial measurement units. Lateral vehicle speed is difficult to measure directly, and indirect calculations are generally carried out using state observers based on kinematic and dynamic models [28]. Roll angle is a physical quantity that describes the degree of inclination of a vehicle in the direction of roll, while pitch angle is the angle that describes the degree of forward and backward inclination of the vehicle. The estimation of vehicle roll angle and pitch angle is one of the important research contents in the field of vehicle attitude control and anti-roll control. Among them, precise modeling methods and complex estimation algorithms are generally the main research directions for improving estimation performance [32]. Vehicle position estimation refers to determining the position of a vehicle in the global coordinate system. The commonly used sensor information for vehicle position estimation includes GPS, inertial measurement units (IMUs), visual sensors, etc. Accurate vehicle position estimation is the core of vehicle navigation, path tracking, and auto drive systems [33].

**Table 1:** From the perspective of estimation objects: overview of vehicle attitude and driving state estimation methods.

Vehicle States	References	Vehicle Relationship of Estimation Objects
Vehicle sideslip angle	[1,9,12,15,23,24,27,31]	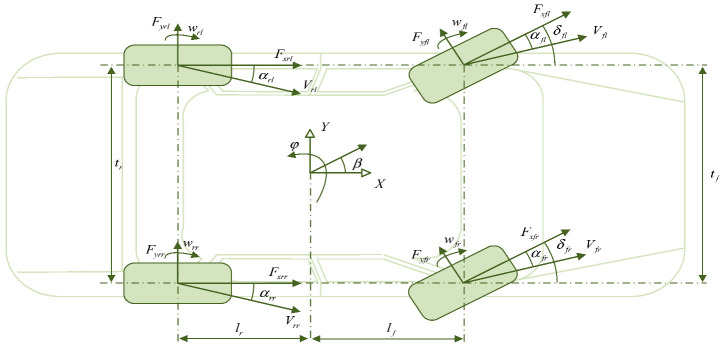 Vehicle state variables including longitudinal, lateral, and yaw degrees of freedom [16].
Vehicle yaw rate	[5,10,14,18,20,22,33]	
Longitudinal and lateral tire force	[2,13,16,19,26,30]	
Longitudinal and lateral vehicle speed	[3,11,29,32]	
Vehicle mass, vehicle roll, and pitch angle	[4,6,7,8,17]	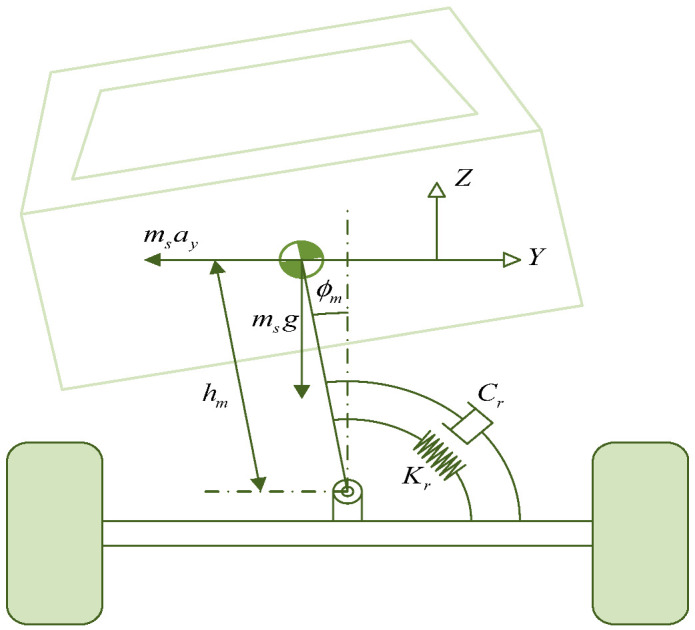 Vehicle state variables including roll dynamics [31].
Vehicle location, road vehicle contact status	[21,25,28]	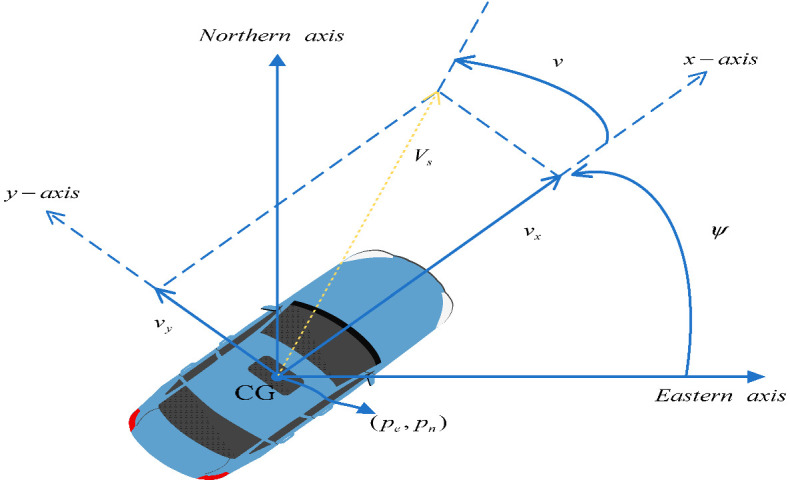 Earth-fixed coordinate system [32].

### 2.2. Estimation of Key Dynamic Parameters of Chassis Components

In addition to the overall driving state of the vehicle, the state estimation of key components of the vehicle chassis is also a focus of research, mainly including tire parameter estimation, steering, driving, braking system state estimation, suspension system state estimation, etc. The status of these key components is directly related to the safety and performance of the vehicle [34,35,36]. By accurately estimating these states, precise control of vehicle movement or precise monitoring of vehicle status can be achieved, ensuring the good operation of the vehicle.

As the only contact component between the vehicle and the ground, the dynamic characteristics of tires directly affect the driving performance and stability of the vehicle. Tire lateral stiffness is one of the key parameters for measuring tire stability and handling performance, which has a significant impact on the suspension system design, handling stability, and driving safety of vehicles [37,38,39,40]. Therefore, accurate estimation of tire lateral stiffness has always been a research hotspot in the field of vehicle engineering. The traditional method for estimating tire lateral stiffness mainly relies on experimental measurements and empirical formulas. However, these methods are often limited by factors such as experimental conditions, measurement accuracy, and tire type, making it difficult to obtain accurate estimation results. In recent years, with the development of sensor technology and mathematical methods, model-based estimation methods have gradually become mainstream. Among them, the least squares method is widely used in tire lateral stiffness estimation due to its simplicity and high estimation accuracy [41,42,43,44]. The recursive least squares method is an improved form of the least squares method, which gradually reduces residuals by iteratively updating parameter values, thereby obtaining more accurate estimation results. In tire lateral stiffness estimations, the recursive least squares method can fully utilize the information of multiple sampling points to improve estimation accuracy and stability. The estimation method of tire sideslip angle has also made significant progress. In estimation methods based on physical models, some studies establish a multi-degree of freedom dynamic model and tire model and use parameters such as front-wheel steering angle information, driving speed, and acceleration to calculate tire sideslip angle. Dasol Jeong et al. [41] designed a state estimator that integrates the bilinear time-varying Kalman filtering algorithm using a vehicle dynamics model and the Dugoff tire model to address the estimation problem of tire sideslip angle and sideslip stiffness.

The estimation of key parameters of the vehicle steering system, driving and transmission system, and braking system is one of the important contents of vehicle chassis dynamics control. The research on key parameter estimation of vehicle steering systems has developed rapidly [45,46,47,48]. Currently, the parameter estimation of steering systems mainly focuses on key parameters such as steering angle and steering torque. Traditional estimation methods are mainly based on empirical formulas and simple sensor data fusion, but these methods have insufficient accuracy and robustness in complex and changing driving environments. Modern estimation techniques, such as Kalman filtering, particle filtering, deep learning, etc., are widely used in steering system parameter estimation to better handle nonlinear and time-varying problems as well as improve estimation accuracy and robustness. In the estimation of key parameters in vehicle drive systems, the focus is on the state parameters of the engine and electric motor [49,50,51]. For example, the engine speed, torque, power, and fuel consumption rate are important indicators that reflect the working status of the engine. For electric motors, it is necessary to estimate their parameters, such as current, voltage, power, temperature, and speed, to monitor the working status and performance of the motor. In terms of transmission system, the key point is on the state parameters of the transmission and transmission shaft [52,53]. The gear position, transmission ratio, oil temperature, and wear condition of the transmission are all key factors in evaluating the performance of the transmission. The torque, speed, and transmission efficiency parameters of the transmission shaft help to understand the working status and efficiency of the transmission system. Jinrak Park et al. [53] proposed a wet clutch actuator piston pressure estimation method and combined it with adaptive control of clutch torque.

The key parameters for the estimation of a braking system are also of great significance, in which the main point is on braking distance, braking deceleration, and the mechanical state of the wheels during braking [54,55,56]. Researchers use various sensors, such as wheel speed sensors and acceleration sensors, to obtain real-time data during vehicle braking and estimate braking system parameters [57,58]. Wei Han et al. [58] established a nonlinear model of the relationship between frictional force and pressure position in an electro-hydraulic braking system and designed a system unknown parameter and pressure estimation method based on a high-order sliding mode algorithm.

The vehicle suspension system, as an important component of the vehicle, plays a crucial role in the comfort and driving stability of the vehicle. The accuracy of its key parameter estimation directly affects the performance analysis and optimization design. Therefore, the study of key parameter estimation for vehicle suspension systems is of great significance [59,60,61,62,63,64,65,66]. Zahra Ahangari Sisi et al. [59] proposed an adaptive adjustment of estimator coefficients and data fusion method for the parameter estimation problem of vehicle suspension system models that effectively improved the accuracy of model construction.

### 2.3. Estimation of Vehicle Driving Environment State

The research on vehicle driving environment state estimation is also of great value in response to the current development trend of electrification, intelligence, and networking of vehicles. This mainly includes information such as road adhesion coefficient, road slope, road type, obstacle spacing, distance between preceding vehicles, and vehicle speed. The effectiveness of driving environment state estimation directly affects the driving safety and stability of intelligent vehicles [67,68].

The road adhesion coefficient is an important parameter that describes the friction characteristics between tires and the road surface and has a significant impact on vehicle driving safety, handling, and economy [42,69,70,71,72]. Therefore, accurate estimation of road adhesion coefficient is crucial for the design of vehicle control systems, especially in complex road conditions where precise estimation has become one of the core technologies for adaptive control of vehicle dynamics.

The current research faces multiple challenges, including the continuous gradient and mutation characteristics of road conditions, strong nonlinearity of vehicle dynamic response, unobservability of small slip rate conditions, and engineering constraints of low-cost sensors and limited computing resources.

From a technical perspective, existing methods can be mainly divided into two categories. Direct estimation based on road surface features extracts physical properties of the road surface using dedicated sensors. Although it has advantages in unstructured roads, it relies on high cost equipment and is susceptible to environmental interference. Indirect estimation based on vehicle response utilizes standard sensors such as wheel speed and inertial measurement units, combined with dynamic models or data-driven methods to infer adhesion coefficients. Due to its cost-effectiveness and ease of deployment, it has become a mainstream solution but is limited by the observation bottleneck in small slip rate areas and the accuracy loss caused by model simplification.

In terms of technological means, current methods include model- and data-driven approaches. The model-driven method embeds the adhesion coefficient into the vehicle dynamics model based on the Kalman filter framework. Although it has high computational efficiency, model mismatch is prone to occur under strong nonlinear operating conditions. The data-driven approach learns mapping relationships directly from sensor data through neural networks, which avoids complex modeling but faces risks of data dependency and overfitting. Multi-source information fusion technology dynamically integrates dynamic models, kinematic models, and heterogeneous sensor data through a layered architecture, significantly improving the adaptability of abrupt road surfaces. Using fusion strategies, the estimation problem of adhesion coefficient and vehicle state parameters can be effectively optimized in synergy, thereby effectively solving the problem of state parameter coupling.

Ehsan Hashemi et al. [42] proposed a robust estimation method for tire force and tire sideslip angle applicable to different road conditions based on vehicle dynamics model and LuGre tire model and verified the reliability of the algorithm. This method is simple and feasible, but its accuracy is influenced by the diversity of road conditions and environmental factors. Within a small slip rate range, the friction coefficient between the tire and the road surface varies linearly with the slip rate. By measuring the slope within this range, the road adhesion coefficient can be estimated. Within a large slip rate range, the friction coefficient between the tire and the road surface tends to stabilize, with a slope close to zero. By measuring the friction coefficient within this range, the road adhesion coefficient can be indirectly estimated. For intelligent driving scenarios, multiple sensor data fitting can be used to estimate the road adhesion coefficient in real time.

The estimation of road slope for vehicle driving has also received the attention of many scholars [73,74]. When the current rear vehicle is on a road with different slopes, unmanned vehicles often find it difficult to effectively obtain accurate vehicle distance. In response to this technical challenge, Zhao Linfeng et al. [73] designed a road slope and distance estimation method by integrating visual and LiDAR information. Lin Gao et al. [74] proposed a multi algorithm fusion method for estimating road slope based on the coupling relationship between heavy vehicle mass and road slope, effectively solving the problem of inaccurate estimation accuracy under sensitive model parameter features.

Intelligent and electrified vehicles are equipped with richer sensors, making new breakthroughs in estimating the state parameters of the driving environment for intelligent vehicles [50,75,76,77]. The estimation and detection of front vehicle speed and distance are crucial for the safe driving of intelligent vehicles. By using the speed sensors carried by the vehicle itself, combined with sensors such as radar and cameras, the driving speed and real-time distance of the preceding vehicle can be detected and calculated in real time. Based on the current position, speed, and acceleration of the vehicle, combined with road features, traffic signals, and surrounding vehicle behavior data, intelligent vehicles can predict their driving trajectory for a period of time in the future. This helps vehicles make decisions in advance, ensuring safety and smoothness of driving. Pengfei Fan et al. [76] proposed a new road level estimation method that considers the difference in fuel consumption rates between flat and graded roads, effectively improving the estimation performance under various operating conditions. Qingxia Zhang et al. [50] proposed a method for estimating road roughness. Based on modeling and parameter identification, an estimation algorithm expression was constructed, and the estimation effect was verified. Rui Song et al. [77] proposed a vehicle attitude fusion estimation strategy for complex working conditions by combining robust observation algorithms, neural network algorithms, and Kalman filtering algorithms to suppress measurement errors in inertial navigation systems.

## 3. Vehicle State Estimation: From the Perspective of Vehicle Characteristics

**Figure 3:**
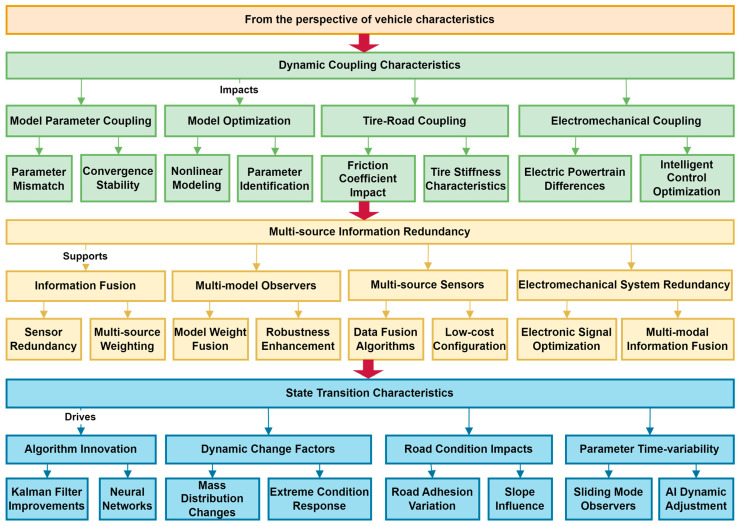
Vehicle state estimation: from the perspective of vehicle characteristics.

The effectiveness of vehicle state estimation is influenced by three core factors: dynamic coupling characteristics, multi-source information redundancy, and state transition characteristics. The dynamic coupling characteristics are reflected in the mutual influence between model parameters, which may lead to model mismatch and algorithm instability. Research on decoupling parameter relationships using fine-grained modeling and multi-model interaction strategies combined with high-precision recognition methods has been performed to improve estimation accuracy. The tire–road coupling characteristics need to be analyzed in conjunction with the reinforcement mechanism of the LuGre tire model, while the electromechanical coupling brought about by electrification needs to be modeled specifically. Multi-source information redundancy is achieved by integrating multiple model observers, multiple sensors, and modal data of electromechanical systems, utilizing complementary redundant information to enhance robustness. In addition, the state transition characteristics focus on the adaptive ability under dynamic operating conditions. The algorithm is required to have dynamic weight adjustment and anti-interference design for sudden changes in road conditions, time-varying parameters, and system interference. The data-driven approach and multi-source information fusion can suppress estimation bias caused by state transitions and support stable output in high-speed/extreme scenarios. The three jointly drive the evolution of vehicle state estimation toward high accuracy, robustness, and real-time performance, providing key support for intelligent driving. The overall research content architecture of this chapter is shown in Figure 3.

### 3.1. Vehicle Dynamics Coupling Characteristics

The coupling characteristics of vehicle dynamics have a significant impact on the effectiveness of vehicle state estimation. In vehicle state estimation, it is usually necessary to establish a vehicle dynamics model to describe the motion state and behavior of the vehicle [78,79,80]. If the coupling characteristics of vehicle dynamics are not fully considered in the model, the model accuracy will be affected, leading to inaccurate state estimation results. Meanwhile, vehicle state estimation algorithms need to face various complex driving scenarios and the uncertainty factors of the vehicle itself. The mismatch of model parameters caused by dynamic coupling characteristics will to some extent affect the robustness of the estimation system. There is a coupling relationship between the parameters of vehicle dynamics models, and a change in one parameter can affect the representation accuracy of other parameters or models. In addition, the coupling relationship between vehicle dynamics model parameters may also lead to the instability of state estimation algorithms [81,82]. The coupling relationship between parameters may prevent the state estimation algorithm from converging to a stable result, or the convergence speed may be very slow. In this case, there may be significant fluctuations and instability in the state estimation results, which can affect the accuracy of vehicle control and decision-making.

To improve the accuracy and stability of vehicle state estimation, it is necessary to fully consider the coupling relationship between parameters in the process of vehicle fine-grained modeling [83,84,85], mainly including selecting appropriate model structures, configuring model parameters reasonably, and so on. The modeling accuracy can be improved by considering nonlinear factors or using high-precision model parameter identification methods. For example, Arash Marashian et al. [79] considered the dynamic linear variation of model parameters and constructed a new model parameter structure using time series modeling. By configuring the model structure and optimizing the model parameters, the accuracy of model parameter identification was effectively improved.

In addition, using appropriate estimation strategies or optimizing estimation algorithms can also handle parameter coupling problems. In practical applications, model parameters and estimation algorithms can be continuously optimized through data-driven approaches, combining experimental data to improve the accuracy and robustness of vehicle state estimation. For example, Yicai Liu et al. [80] considered the parameter coupling relationship between car mass, road slope, and vehicle roll angle and proposed an adaptive estimation strategy based on multi-model interaction and parameter dynamic adjustment. Using optimization algorithms and coupled estimation strategies, the estimation accuracy in the current situation can be improved.

The impact of vehicle dynamics coupling characteristics on estimation systems involves the motion patterns of vehicles under different road and driving conditions, as well as the interaction between tires and the ground. At the same time, it is necessary to consider the coupling relationship between the vehicle kinematic model and the dynamic model, as well as the coupling relationship between the vehicle dynamic model parameters and chassis components. Kay Uwe Henning et al. [81] integrated vehicle state and parameter estimation methods into the vehicle motion control strategy, considering the model coupling relationship between vehicle motion state and road state. Based on nonlinear models and Kalman filtering, the estimation effect was improved. Vicent Rodrigo Marco et al. [83] considered the factors of model parameter coupling uncertainty and observation noise and adopted a multimodal information fusion estimation scheme and an adaptive estimation algorithm to improve the reliability of the estimation system. Xu Li et al. [85] designed a vehicle kinematic model observer and a dynamic model observer, utilizing redundant features of sensor information and various improved Kalman filtering algorithms to improve the accuracy of the state estimation system using observer-weighted optimization and dynamic fusion.

The coupling characteristics of tires and roads, as well as the parameter relationship between tire road contact surfaces, are important aspects of current research on vehicle state estimation. As the only contact point between the vehicle and the ground, the characteristics of tires have a significant impact on the vehicle’s dynamic characteristics. The friction coefficient and lateral stiffness of tires are directly related to the driving stability and handling of vehicles. The changes in tire characteristics will directly affect the dynamic behavior of vehicles under different road conditions and driving scenarios. For example, on slippery roads, the friction coefficient of the tires will decrease, leading to an increase in the braking distance of the vehicle and increased difficulty in handling. The in-depth study of tire characteristics using parameter identification reveals its impact mechanism on vehicle dynamics, which is of great significance for improving the effectiveness of vehicle state estimation [36,86,87,88].

With the rapid development of intelligence and electrification technology, the influence of vehicle dynamics electromechanical coupling characteristics on vehicle state estimation cannot be ignored. The power transmission mechanism of electric vehicles is significantly different from traditional fuel vehicles, and the chassis steering, braking, and suspension systems also show a trend of electromechanical coupling and electric integration with the development of wire control. Intelligent driving technology and chassis electric integration technology provide richer and more redundant information for vehicle state estimation systems; however, at the same time, they also increase the degrees of freedom of vehicle dynamics systems and set higher standards for vehicle dynamic response and performance requirements [63,89,90]. The innovative chassis carrier contains more complex dynamic coupling characteristics, and the vehicle dynamics system oriented toward intelligence and electrification also relies more on the accuracy and reliability of state signal acquisition. Future research on vehicle state estimation needs to pay more attention to new characteristics and problems in the context of intelligence and electrification, providing strong support for the sustained development of the field of vehicle engineering.

### 3.2. Redundancy Characteristics of Vehicle Multi-Source Information

The redundancy characteristics of vehicle multi-source information mainly include the redundancy characteristics of vehicle multi-model observer information [77,91,92,93,94,95,96], vehicle multi-source sensor information redundancy [90,97,98,99,100,101,102,103], and vehicle electromechanical coupling system multimodal information redundancy [104,105,106,107,108,109,110]. For example, Francesco Tufano et al. [96] proposed an interactive multimodal state observer fusion estimation method. They designed state observers using extended Kalman and unscented Kalman algorithms to improve the accuracy and robustness of state estimation methods under complex road conditions, respectively. Xin Xia et al. [97] fused redundant multi-source sensor information, such as inertial measurement units and global navigation satellite system information, and proposed a vehicle centroid sideslip angle estimation method based on the Kalman filtering algorithm. B.L. Boada [108] et al. effectively utilized vehicle multimodal information and treated neural network estimates as virtual sensor data, constructing a robust observer based on energy peak filtering and neural networks.

The redundancy characteristic of multi-model observer information in vehicle state estimation refers to the use of multiple different vehicle models or observation methods to calculate the vehicle state, and there is redundancy between these observer information. Using this information redundancy feature to achieve optimized estimation, the accuracy, anti-interference ability, and adaptability of vehicle state estimation can be further enhanced [77,91,92,93,94,95,96]. Different vehicle model observers may have different characteristics and advantages for vehicle dynamics and kinematic model mechanisms, driving conditions, nonlinearity, and other characteristics. Observers based on different vehicle mechanism models have different applicability and accuracy in different vehicle states and operating conditions. For example, observers based on kinematic models have better estimation performance when the nonlinearity of vehicle dynamics is weak and the dynamic change characteristics are fast. The observer based on dynamic models has better estimation performance when the vehicle has strong nonlinear dynamic characteristics and the state changes tend to be stable. By fusing the information of multiple models, the advantages of each model can be used to make up for the shortcomings of a single model, so as to achieve more accurate state estimation in various environments and road conditions. How to optimize the allocation of weights for multiple model observers based on vehicle driving conditions and real-time status is the focus of estimation strategy design. For example, Jiwon J. Oh et al. [91] designed a vehicle multi-model observer using redundant sensor information collected from inertial measurement units and onboard sensors, combined with a monorail vehicle model and a dynamic model. The estimation performance was improved by dynamically weighting the redundant observer information. Meanwhile, when certain models are affected by factors such as noise, interference, or model mismatch, their estimation results may deviate from the true values. By integrating information from multiple model observers, the redundancy of observation information can be utilized to reduce the impact of adverse factors and improve the robustness of the estimation system. Even if a single observer experiences errors, other model observers can still provide effective information about the vehicle’s state, ensuring that the state estimation results do not experience abrupt changes. Y.H. Liu et al. [92] constructed an iterative state estimation system by combining particle filtering and extended Kalman algorithm, achieving optimization of the estimation system.

The redundancy characteristics of multi-source sensor information in vehicles also have a significant impact on the effectiveness of vehicle state estimation. Multi-source sensor information redundancy refers to obtaining information about vehicle status and environment through multiple sensors. There is redundancy between this information, that is, different sensors may provide multiple pieces of information about the same status or environmental characteristics [90,97,98,99,100,101,102,103,104,105]. Information can be fused using data fusion, filtering algorithms and other technologies to obtain more accurate and reliable state estimation results. For example, Xin Xia et al. [97] integrated redundant sensor information, such as IMU and GPS, and improved the accuracy of vehicle centroid sideslip angle estimation using error dynamics model construction and redundant information processing.

Meanwhile, utilizing the redundant characteristics of multi-sensor information can also enhance the robustness of vehicle state estimation systems. Considering the signal fluctuation and misalignment issues that may exist with a single sensor, as well as the multi-scenario applicability issues that vehicles need to consider when facing a wide range of complex working conditions, an estimation strategy based on multi-sensor information redundancy can significantly improve the accuracy and stability of the estimation system. For example, B.L. Boada et al. [98] obtained information on vehicle lateral acceleration, vehicle speed, yaw rate, and steering angle and used a combination of neural networks and Kalman filtering algorithm to construct an estimation system, improving the reliability of the observation system by processing redundant data.

The redundancy of multi-source sensor information can also improve the computational efficiency and dynamic observation ability of vehicle state estimation systems. Using the vehicle state information provided by multiple sensors and optimizing the estimation strategy and architecture design, parallel processing of redundant data can be achieved, which can effectively simultaneously improve the efficiency of state estimation and improve real-time performance. For example, A.C. Rafael et al. [101] utilized the redundancy advantage of in vehicle sensor information to obtain multi-dimensional tire force estimation results, and used delayed interconnection to solve the problems of interdependence and easily affected estimation efficiency in the cascaded estimation architecture.

In the context of electrification and intelligence, the comprehensive optimization and utilization of multimodal redundant information in-vehicle electromechanical coupling systems is also of great significance for improving the effectiveness of vehicle state estimation [104,105,106,107,108,109,110]. Compared to traditional fuel vehicles, the chassis system of electric vehicles shows a trend toward line control, intelligence, and modularity. It tends to use electric motors to replace complex mechanical structures, and the demand for signal interaction and decision fusion scenarios between different chassis modules is becoming increasingly evident. Therefore, under the trend of electrification and intelligence development, the vehicle chassis usually needs to be equipped with redundant sensing devices to improve the accuracy of information acquisition and fault tolerance in the face of interference and faults through the fusion of redundant signals. Under these conditions, by utilizing the redundant nature of information, identical or similar information can be obtained from multiple sources, thus enabling a more accurate and reliable approximation of the true vehicle state. Meanwhile, for intelligent electric vehicles, the state information carried by electric drive signals has a significant advantage of fast response. By utilizing the redundancy of information, the speed of data processing and state acquisition can be accelerated, thereby improving the dynamic response capability and real-time performance of the vehicle state estimation system. For example, W. Liu et al. [106] proposed a high-precision closed-loop iterative estimation method for the state of four-wheel independent drive electric vehicles, utilizing the redundant characteristics of electromechanical drive information and multi-model Kalman filtering.

Meanwhile, the development of intelligent technology enables vehicles to obtain richer vehicle status information using various sensors and actuators. For example, using radar, cameras, LiDAR and other devices, it is possible to obtain information about the surrounding environment of vehicles, including road conditions, traffic conditions, the location of pedestrians and other vehicles. These pieces of information, combined with electromechanical coupling information, can provide a more comprehensive estimation of the vehicle’s state and provide more accurate data support for advanced driving assistance systems, such as autonomous driving.

The configuration method of redundant information of intelligent electric vehicles is also of great value in reducing the design cost of estimation systems. Through the redundant configuration of multiple low-cost sensors, vehicle status information can be obtained from multiple perspectives and dimensions, thereby gaining a more comprehensive understanding of the vehicle’s operating status. This redundant configuration can compensate for potential errors or deficiencies in individual sensors, improving the accuracy and reliability of vehicle state estimation. A low-cost sensor redundancy configuration means that in the event of some sensors malfunctioning or failing, relevant information can still be obtained from other sensors, thereby maintaining the stability and continuity of vehicle state estimation and aiding in system maintenance. Compared with high-performance and high cost complex precision sensors, low-cost sensors are easier to achieve redundant configurations and utilize the redundancy of multi-source sensor information in intelligent electric vehicles to improve estimation performance. With reasonable selection and design, the cost of the entire vehicle state estimation system can be reduced while ensuring estimation accuracy and reliability. For example, A.K. Madhusudhanan et al. [104] utilized a new low-cost load sensing bearing for tire force estimation, avoiding the use of expensive six component force sensors. X.L. Ding et al. [109] utilized redundant low-cost information for critical vehicle driving state estimation and ensured good state estimation performance by optimizing estimation strategy design and developing critical estimation algorithms.

### 3.3. Vehicle State Transition Characteristics

The impact of vehicle state transition characteristics on the effectiveness of vehicle state estimation cannot be ignored under large-scale and complex road conditions. The state of a vehicle is not static and is influenced by various factors, including the dynamic characteristics of the vehicle itself, the driving behavior of the driver, road conditions, and dynamic changes in the external environment. The characteristics of vehicle state transition composed of these factors will directly affect the effectiveness of vehicle state estimation [41,111,112,113,114,115]. The dynamic characteristics of a vehicle itself are a key factor affecting its state transition. Due to differences in structure, mass, suspension system, power system, and other aspects, different vehicles may have different dynamic characteristics. In this case, a single estimation algorithm is often difficult to apply to all vehicle models and driving conditions. Especially at high speeds and under extreme operating conditions, the rate of vehicle state change is more rapid and complex, and the impact of dynamic transition characteristics on state estimation performance will become more apparent.

The dynamic changes in road conditions are also an important factor affecting the changes in vehicle status. The changes in road surface grade and adhesion coefficient directly affect the vehicle’s motion state. Therefore, the dynamic adaptive ability and robustness of vehicle state estimation systems in the face of various complex working conditions and vehicle state transitions are important indicators for evaluating the quality of estimation strategies. T. Chen et al. [113] proposed an adaptive fusion estimation strategy for vehicle states under large-scale complex working conditions. They designed vehicle state Kalman filters based on vehicle kinematic equations, dynamic equations, linear tire models, and nonlinear tire models and then dynamically adjusted the weights of different observers based on real-time vehicle state characteristics, significantly improving the estimation accuracy and adaptive ability of the estimation system under different working conditions.

The time-varying characteristics of system parameters, model uncertainty, and system interference have been the focus of attention for many scholars on the effectiveness of vehicle state estimation [42,116,117,118,119,120,121,122]. Model uncertainty and interference mainly come from modeling errors in control systems, the system itself, and external disturbance signals, which are the main reasons for the decrease in accuracy of model-based observers. At the same time, uncertainty and interference can also lead to a decrease in the robustness of the estimation, resulting in phenomena such as bias, lag, and fluctuations in the estimation results. Therefore, in order to improve the effectiveness of vehicle state estimation, the transition characteristics of vehicle states are an important factor affecting observation accuracy in the process of vehicle dynamic modeling and estimator design. Cuauhtémoc Acosta Lúa et al. [115] considered the model disturbances and parameter uncertainties induced by the driving environment and constructed a high-order sliding mode algorithm based on a state observer to improve the estimation performance under dynamic state transitions. T. Chen et al. [116] accurately distinguished the difference between heading angle and yaw angle during the state transition process of intelligent vehicles and constructed a model. They designed a state observer with constrained stability, effectively improving the robustness of the estimation results. The matching design of optimization algorithms is an important way to solve system uncertainty and interference in the estimator design process, and it is also crucial for improving the effectiveness of vehicle dynamics control. At the same time, combining redundant sensors for multi-source information fusion can also reduce the uncertainty and interference faced in the process of state transition. In recent years, with the development of data-driven and artificial intelligence algorithms, it can assist in real-time updating and dynamic adjustment of vehicle model parameters and also contribute to the improvement of estimation performance under state transition situations.

## 4. Vehicle State Estimation: From the Perspective of Key Algorithms

**Figure 4:**
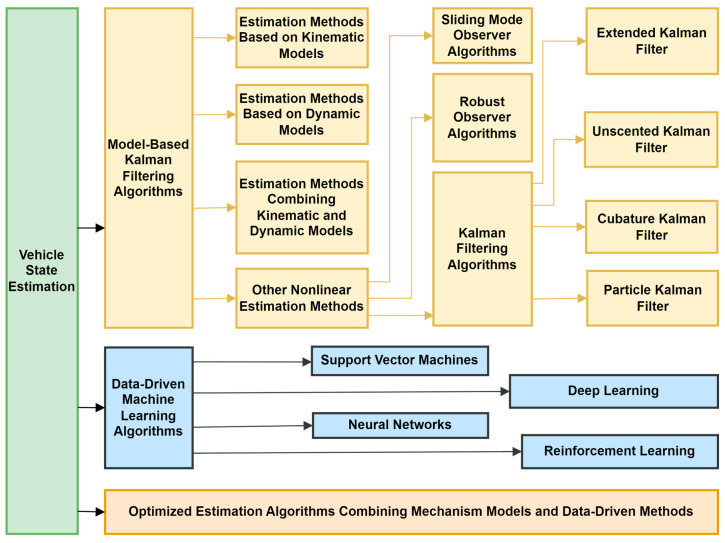
Vehicle state estimation: from the perspective of key algorithms.

The key algorithms for vehicle state estimation are mainly divided into three categories: model-based Kalman filtering, data-driven machine learning algorithms, and fusion optimization methods combining the two. The model-based Kalman filtering algorithm focuses on vehicle kinematics and dynamics models and combines state observers with dynamic equations to achieve dynamic behavior description. The kinematic model is suitable for low-speed simple scenarios, relying on parameters such as speed and steering angle. The dynamic model analyzes the force relationship under complex working conditions using nonlinearity, tire characteristics, and other factors. The Kalman filter optimizes noisy data using a prediction update recursive framework, and its improved form utilizes linearization or probability sampling to handle nonlinear problems, improving the estimation accuracy of high-dimensional state spaces. Data-driven machine learning algorithms utilize deep learning, neural networks, support vector machines, and reinforcement learning to parse complex sensor data. However, data quality and annotation accuracy have a significant impact on model performance, and complex models require efficient training algorithm support. The fusion optimization algorithm combines the advantages of model and data-driven approaches, providing an interpretable framework based on physical meaning mechanism models. The data-driven method optimizes model parameters and compensates for mismatches in complex operating conditions. This type of fusion method improves robustness through parameter collaboration, weight adaptation, and other means and has become a current research hotspot. However, its performance depends on the balanced design of data preprocessing and algorithm selection strategies. The overall research content architecture of this chapter is shown in Figure 4.

### 4.1. Model-Based Kalman Filtering Algorithm

The most commonly used approach is to design a vehicle state observer based on vehicle mechanism models and dynamic equations, matched with appropriate estimation algorithms. The model-based vehicle state estimation method can accurately describe the dynamic behavior and dynamic coupling relationship of vehicles in actual operation and has been widely verified to have good estimation effects in various application scenarios. In model-based vehicle state estimation methods, there are mainly estimation methods based on kinematic models, estimation methods based on dynamic models, estimation methods that integrate kinematic and dynamic models, and other nonlinear estimation methods [1,2,4,5,6,8,9,10,112,123,124,125]. The overview of vehicle state estimation methods from different perspectives is listed in Table 2.

The kinematic model mainly focuses on external motion parameters such as vehicle speed, acceleration, and steering angle. By combining sensor data such as vehicle wheel speed and steering angle with the vehicle’s kinematic model, vehicle motion state estimation can be achieved, which is usually suitable for vehicle state estimation under low speed and simple road conditions. The dynamic model considers the dynamic characteristics of vehicles, such as nonlinearity, tire characteristics, and parameter coupling, and can more accurately characterize the force situation and dynamic relationship of vehicles during operation. It is usually suitable for vehicle state estimation under complex road conditions such as high speed, strong nonlinearity, and sharp turning. Both kinematic model-based observation methods and dynamic-based observation methods have corresponding advantages and applicable conditions. Combining kinematic and dynamic models can fully leverage their advantages and improve the accuracy and robustness of vehicle state estimation. The optimal estimation effect can be achieved through the fusion of multi-sensor data or multiple model observers, optimization of state estimation algorithms, and other technical means.

The dynamic relationship of vehicles in actual operation is often nonlinear, so the application of nonlinear observation theory in vehicle state estimation research is also very extensive. Common nonlinear state estimation algorithms include sliding mode observation algorithms [5,20,62], robust observation algorithms [1,15,42,58,79,82], and Kalman filtering algorithms. Among them, the Kalman filtering algorithm [12,13,14] is the most widely used, mainly including the extended Kalman filter [12,72,106], unscented Kalman filter [21,32,98], adaptive extended Kalman filter [8,80], cubature Kalman filter [77,107,112,113], and particle Kalman filter [92,124]. These methods can handle uncertainty and noise in nonlinear systems, improving the accuracy and stability of state estimation.

Kalman filtering is an efficient and recursive state estimation algorithm. It relies on two core steps of prediction and update and obtains the optimal state estimation at the current moment by weighted fusion of the previous moment’s state estimation and the current moment’s observation data. Kalman filtering can effectively process measurement data with noise and optimize state estimation results by minimizing the covariance of estimation errors.

In the iterative step of Kalman filtering, the filter needs to be initialized first to determine the initial state estimation value *x_0_* and the initial estimation error covariance matrix *P_0_*. *x_0_* is a prior estimate of the initial state of the system, and *P_0_* reflects the degree of uncertainty in the initial state estimation. The prediction steps include state prediction and error covariance prediction. The state transition equation can be expressed as *x_k_* = *F_k_x_k-1_* + *B_k_u_k_* + *w_k-1_*, where *x_k_* is the state vector at time *k*, *F_k_* is the state transition matrix, *B_k_* is the input data, *u_k_* is the control variable, and *w_k-1_* is the process noise. The update formula for the covariance matrix of prediction and estimation errors is *P_k_* = *F_k_P_k-1_F^T^_k_* + *Q_k-1_*, where *P_k_* represents the prior estimation error covariance matrix and *Q_k-1_* is the process noise covariance matrix. The update step includes a Kalman gain calculation and state update. The Kalman gain *K_k_* determines the weight of the measurement information when updating the state estimation. The calculation formula is *K_k_* = *P_k_H^T^_k_*(*H_k_P_k_H^T^_k_* + *R_k_*)^−1^, where *H_k_* is the observation matrix. In the update of error covariance, the calculation formula is *P_k_* = (*I*-*K_k_H_k_*)*P_k_*, where *I* is the identity matrix. The Kalman filter algorithm recursively calculates the Kalman gain, state updates, and estimation error covariance update equations and uses new measurement information to correct the state estimation at each time step, so that the estimation results can best approximate the true state.

Extended Kalman filtering is an extended form of Kalman filtering used to handle nonlinear vehicle dynamic models. It approximates nonlinear models to linear models using local linearization techniques and performs linear Kalman filtering at each time step. This method not only retains the computational efficiency of Kalman filtering, but also improves the adaptability to nonlinear vehicle dynamic models. Filtering algorithms such as unscented Kalman filtering and volumetric Kalman filtering are essentially improved forms of extended Kalman filtering. UKF processes nonlinear vehicle dynamic models through lossless transformation, improving estimation accuracy and stability. On the basis of UKF, volumetric Kalman filtering can further overcome the possible divergence or accuracy degradation of UKF in high-dimensional state space. Particle filtering is a nonlinear filtering method based on Bayesian estimation. It approximates the posterior probability distribution of the vehicle’s state through a set of random samples (particles). The improved form of filtering algorithm can handle non-Gaussian and nonlinear vehicle dynamic models, demonstrating better estimation performance in complex environments.

### 4.2. Data-Driven Machine Learning Algorithm

Data-driven machine learning algorithms, such as deep learning, support vector machines, neural networks, etc., have strong nonlinear mapping capabilities and adaptability, and can learn complex patterns from large amounts of sensor data, thereby improving the accuracy and robustness of state estimation. Data-driven machine learning algorithms have a wide range of applications in vehicle state estimation [32,33,46,47,48,49,50,56,64,69,70,71], which can not only improve the accuracy and robustness of state estimation, but also handle complex sensor data and environmental noise. Common data-driven algorithms include support vector machines, deep learning, neural networks, reinforcement learning, and so on.

The application of machine learning algorithms in the field of vehicle state estimation has become a current research hotspot [126,127,128,129]. Deep learning is a branch of machine learning algorithms that enable computers to learn and understand complex data by simulating the workings of human brain neural networks. In vehicle state estimation, deep learning can process and parse complex sensor data to obtain more accurate state estimation results. In addition, deep learning can also handle noise and interference in the environment, improving the robustness of state estimation. Deep learning models typically consist of input layers, hidden layers, and output layers. In vehicle state estimation, the input layer can receive sensor data from the vehicle, while the output layer outputs the vehicle’s state estimation results. Through extensive data training, deep learning models can gradually optimize model parameters to improve state estimation accuracy. In addition, various optimization techniques such as regularization and normalization as well as different optimization algorithms such as stochastic gradient descent and Adam can also be used to find the optimal solution [130,131]. Neural networks, as an important branch of machine learning, have shown strong potential in vehicle state estimation. It constructs a complex network structure by simulating the connection of human brain neurons, thereby achieving nonlinear mapping of input data. In vehicle state estimation, neural networks can extract key features from multi-source sensor data and output accurate vehicle state estimation values through learning and training. For example, Pengfei Fan et al. [128] constructed an engine output power estimation model using artificial neural network algorithms and long short-term memory convolution algorithms and verified that the algorithm can significantly improve estimation accuracy and stability under multiple operating conditions using quantitative statistics. S. Sasikala et al. [132] proposed a perceptual spatiotemporal multi-scale network estimation approach that improved the overall performance of vehicle and traffic volume estimation.

In addition to neural networks and deep learning techniques, support vector machines (SVMs) have also been effectively applied in vehicle state estimation. SVMs maximize the boundaries between different categories by solving the optimal hyperplane, thereby achieving classification or task regression. In vehicle state estimation, SVMs can effectively handle various complex nonlinear problems, and their powerful generalization ability enables the model to maintain high performance on new data. Shunli Wang et al. [133] proposed a lithium-ion battery state estimation method that takes into account the time-varying characteristics of model parameters. They constructed an adaptive radial basis support vector machine model and used the minimum quadratic algorithm to achieve state of charge estimation under time-varying parameters, significantly improving the robustness of the estimation system. Reinforcement learning learns the optimal decision strategy through trial and error, enabling the model to gradually learn the optimal estimation strategy through interaction with the environment. In vehicle state estimation, reinforcement learning algorithms can adaptively adjust estimation strategies to cope with various complex and variable environmental conditions [132,133,134,135,136], enabling the model to maintain high estimation accuracy in different scenarios. It is worth noting that in the practical application of machine learning algorithms, the impact of data quality and annotation accuracy on model performance, estimation performance, and estimation credibility cannot be ignored. How to obtain high-quality and accurately labeled datasets in vehicle state estimation is a worthwhile research topic. With the increasing complexity of deep learning models, designing more efficient training algorithms and model optimization strategies is also an important research direction [137,138,139]. For example, Viktor Rakhmatulin et al. [136] combined deep neural network estimation methods with automatic data annotation methods to address the problem of complex physical model features of the parameters to be estimated. They enhanced the accuracy, reliability, and generalization ability of deep learning algorithms by using automatic annotation of training data.

### 4.3. Optimization Estimation Algorithm Combining Mechanism-Based Models and Data-Driven Approaches

With the improvement and complexity of vehicle control system functionality, as well as the increasing demand for precision in vehicle motion collaborative control under the trend of electrification and intelligence, existing research has put forward higher requirements for the accuracy, robustness, real-time, and other comprehensive performance of vehicle driving state estimation. It is difficult for state estimation methods with a single structure and simple principles to fully meet existing requirements. The mechanism model is established based on physical laws and vehicle dynamics principles, and the parameters have clear physical meanings [30,33,35]. The model parameters are easy to adjust and have strong adaptability. However, for certain complex working conditions, the mechanism model may be difficult to accurately describe the behavior of the vehicle, and model-based estimators may experience a decrease in accuracy due to model mismatch. The data-driven approach utilizes large-scale statistical data and discovers intrinsic relationships between data through heuristic rules or deep learning models. This method has strong modeling ability for complex nonlinear systems, but usually requires a large amount of data support [46,47].

The estimation methods based on mechanistic models and data-driven approaches, as two different forms of state estimation, each have their own advantages and disadvantages. If the advantages of the two methods can be combined, the effectiveness of vehicle state estimation can be further improved [137,138,139,140,141,142,143,144,145,146,147,148], which has important research value.

By combining the mechanism model and data-driven vehicle state optimization estimation algorithm, the vehicle motion equation is established using the mechanism model. Then, the model parameters are optimized using data-driven methods, which can effectively improve the accuracy of state estimation [49,50,51,54,55]. The parameters in the mechanism model usually have clear physical meanings but may be difficult to accurately determine in practical applications. Using data-driven methods, model parameters can be optimized and adjusted using actual observation data to improve the accuracy of the model [144,147,148]. Common algorithms used include least squares, Kalman filtering, genetic algorithms, etc. By combining mechanism models and data-driven methods, the algorithm can better adapt to vehicle state estimation under complex working conditions. Based on different data characteristics, model advantages and disadvantages, and vehicle dynamic changes, the model weight can be adaptively adjusted to achieve more accurate vehicle state estimation. For example, Xinwei Yang et al. [17] proposed a comprehensive vehicle model and data-driven form of state estimation architecture, which updates and improves model parameters using data-driven algorithms to achieve improved estimation performance. Haikuan Lu et al. [143] designed a Kalman filtering algorithm based on vehicle models and matched it with a generalized pseudo Bayesian algorithm to achieve mobile horizon estimation. The effectiveness of the proposed method was verified using multi-scenario simulation testing. Vedhanayaki Selvaraj et al. [144] proposed a hyperparameter selection method based on a Bayesian optimization algorithm to improve the performance of machine learning models and achieve the selection of optimal hyperparameters. They compared and tested the performance of various optimization algorithms in hyperparameter adjustment. Mostafa Rahimi et al. [148] proposed a neural network modeling method based on the establishment of a vehicle dynamics model and used bench test data for model training and validation, thereby significantly improving the modeling and estimation performance.

The fusion optimization algorithm combines the advantages of models and data-driven methods, utilizing both the interpretability framework of mechanism models and data-driven methods to optimize model parameters, compensating for the problem of model mismatch under complex operating conditions and improving the accuracy and robustness of state estimation. However, this method often relies on the quality of data preprocessing and the rationality of algorithm selection and needs to seek a balance between the two to achieve optimal results. The comparison between different estimation algorithms is shown in Table 3. The selection and application of optimization estimation algorithms need to be balanced based on model characteristics. For example, for situations where the model is simple and requires high real-time performance, it is necessary to choose algorithms with faster convergence speed but slightly weaker search ability. For complex models with high accuracy requirements, it is necessary to choose algorithms with strong search capabilities but high computational complexity. It is also possible to consider combining different optimization algorithms to fully utilize their respective advantages and further improve the performance of vehicle state estimation [59,60,80]. In addition to the selection and application of the algorithm itself, factors such as data quality, model structure, and algorithm parameters can also affect the performance of vehicle state estimation. When applying optimization algorithms for vehicle state estimation, it is also necessary to comprehensively consider these factors and carry out corresponding preprocessing and optimization work [139,140,141,142,143,146].

## 5. The Main Application Scenarios of Vehicle State Estimation Technology

### 5.1. Application in Vehicle Autonomous Driving Scenarios

Vehicle state acquisition is one of the important contents in autonomous driving technology. In the existing research, most methods provide accurate positioning and navigation information, body posture, stable driving state, and other key information for autonomous vehicles by fusing multiple sensor data, so that vehicles can achieve accurate positioning, reasonable path planning, precise dynamic coordination control, and other functions in complex driving scenes, thus ensuring the good operation of the auto drive system [33,35,36,43,45,46,47,48,49,51,68,69,70,71].

The main purpose of vehicle state estimation is to provide accurate and reliable signal feedback inputs for decision-making planning and precise control of intelligent vehicle motion [50,76,77,78,100]. In the vehicle auto drive system, an accurate estimated vehicle state can effectively control the acceleration, deceleration, steering, and other operations of the vehicle, so as to achieve the driving goals of vehicle obstacle avoidance and trajectory tracking as well as significantly improve the stability and safety of the vehicle during driving. In addition, vehicle state estimation technology can also provide key data information for the behavior decision-making and path planning of the auto drive system through real-time perception of the vehicle’s own state and surrounding environment information. By continuously optimizing the algorithm and improving the sensor accuracy, the accuracy and real-time of vehicle state acquisition can be further improved, thus improving the overall performance of the autonomous driving system [113,119,125,126].

The application research of vehicle state estimation in auto drive systems has a broad application prospects and has been practiced in many related researches at present. Under typical road conditions, corresponding state estimation methods can provide accurate key state information such as vehicle speed, acceleration, and distance between preceding vehicles. Combined with road conditions and traffic signal data, they can provide the optimal driving path and strategy for the vehicle. Ying Zhang et al. [69] proposed an optimization algorithm for estimating road vehicle density using drone camera information and constructed a multi-scale perception vehicle density estimation network. Pragyan Dahal et al. [70] proposed an intelligent vehicle motion predictor based on recurrent neural networks that significantly improves the accuracy and robustness of vehicle state estimation. Yan Wang et al. [125] proposed a state estimation method based on Kalman filtering and interaction models to address the transmission interval, bandwidth limitations, and inapplicability to a wide range of driving conditions in V2V communication, effectively improving the estimation performance and adaptability under communication rate constraints. With the continuous development and popularization of automatic driving technology, vehicle state estimation technology will be fully integrated into the auto drive system and play an important role [145,146,147]. At the same time, vehicle state estimation technology can be deeply integrated with high-precision maps, traffic signal control, internet of vehicles and other technologies, so as to improve the estimation accuracy and provide reliable information input for the autonomous driving system [149,150].

The integration of vehicle state estimation technology in actual vehicle systems has made certain progress. Many car manufacturers and research institutions are actively exploring how to apply advanced state estimation algorithms to actual vehicles. Some high-end car models have begun to adopt state estimation techniques based on Kalman filtering and particle filtering for their autonomous driving assistance systems. These systems integrate data from various sensors such as GPS, IMU, cameras, and LiDAR to achieve real-time estimation of vehicle position, speed, attitude, and other states, providing reliable data support for autonomous driving functions.

At present, some mass-produced autonomous driving vehicles have integrated vehicle state estimation technology using the following methods: First, high-precision sensor combinations, such as RTK-GPS and vehicle grade IMU deep integration, have been used for real-time estimation of vehicle position and attitude with the Kalman filtering algorithm, providing centimeter-level positioning data for vehicle path planning and decision-making systems. For example, Waymo’s autonomous vehicles are equipped with multiple laser radars, cameras, and millimeter wave radars, combined with high-precision maps, using multi-sensor fusion algorithms to achieve accurate perception of the surrounding environment of the vehicle. The dynamic driving status of the vehicle is also estimated using vehicle state estimation algorithms, such as lateral and longitudinal speed, acceleration, etc., to achieve safe and smooth autonomous driving. Second, based on the data exchange of the vehicle bus system, the vehicle state estimation module transmits real-time data to the autonomous driving controller, power system controller, etc. through the controller area network (CAN) bus, ensuring timely updates and sharing of vehicle state information. At the same time, in response to issues such as sensor failures in practical applications, redundant design and fault diagnosis algorithms are adopted. When the main sensor fails, it can quickly switch to the backup sensor, ensuring the continuity and reliability of vehicle state estimation.

### 5.2. Application in Vehicle Active Safety Control

Vehicle state estimation technology plays a crucial role in modern vehicle active safety systems. Accurate and reliable state estimation technology can monitor the driving status and surrounding environment of vehicles in real-time, providing a timely and accurate decision-making basis for collision warning and avoidance systems, driver assistance systems, and stability control systems [6,17,21,25,151,152,153,154]. By accurately obtaining key state parameters such as vehicle speed, acceleration, steering angle, and yaw rate and combining various sensor data such as high-precision maps, radar, and cameras, vehicle state estimation technology can construct a three-dimensional motion model of the vehicle, achieving comprehensive perception of the vehicle’s dynamic behavior [28,30,31].

Research on the combination of state estimation and vehicle stability control is the most common [46,155,156]. By combining accurate and reliable vehicle state estimation results, real-time vehicle state changes can be provided for the active safety control system of vehicles, thereby providing key data support for stability control decisions [38,43,45,46,47,48,52,53,54,55,56,57,58,60,61,62]. By comparing the differences between the actual vehicle state and the ideal state, the control system can adjust the vehicle control parameters or control laws in a timely manner, such as front wheel steering angle, driving force, braking force, yaw moment, anti-roll moment, etc., to ensure the stability of the vehicle under complex or extreme working conditions such as severe steering, high-speed driving, emergency braking, etc. Good state estimation performance plays an important role in improving vehicle handling and stability, reducing accident risks such as sideslip and rollover. Xinwei Yang et al. [17] designed a vehicle roll angle state estimator, center of gravity height, roll moment of inertia, and corresponding suspension parameter estimator to address the technical challenges of high center of gravity, large volume, and easy rollover of heavy-duty vehicles, effectively improving the transient response and dynamic prediction ability of vehicle dynamics control. Wei Han et al. [58] considered the nonlinearity and uncertainty of electro-hydraulic braking systems and designed an adaptive sliding mode hydraulic control method based on hydraulic estimation that eliminated the negative effects of model parameter uncertainty and interference. Nils Pletschen et al. [151] proposed a new nonlinear estimation method for suspension parameters by combining the Kalman filtering algorithm and Takagi Sugeno modeling theory, effectively improving the performance of suspension in both passive and active control. Qian Lu et al. [119] proposed an active integrated control method for sideslip and yaw stability based on vehicle center of mass sideslip angle estimation, which effectively improves the stability and handling of vehicles under extreme turning conditions.

Many studies also focus on the application of state estimation technology in collision warning and avoidance systems. Vehicle state estimation technology can issue timely warnings when potential collision risks arise and assist vehicles in autonomous obstacle avoidance by analyzing real-time vehicle trajectory, speed, and surrounding environmental information [119,122]. The implementation of collision avoidance function not only relies on accurate and dynamic acquisition of vehicle real-time status, but also requires the combination of advanced algorithms and models for risk assessment and decision-making. The accuracy and real-time performance of state acquisition directly affect the operational effectiveness of the system, which is of great value in reducing potential collision risks and improving driving safety [139,147,157,158].

With the continuous optimization and improvement of vehicle control systems, vehicle state estimation technology can play a more important role in active safety control of vehicles in intelligent driving scenarios. Through the comprehensive interconnection among vehicles, between vehicles and roads, and between vehicles and clouds, vehicles will be able to obtain more comprehensive status information of surrounding vehicles and roads in real time. By combining with technologies such as the internet of vehicles, cloud computing, and big data, the vehicle state estimation system has a richer source of information, higher redundancy of information, and greater optimization space for improving estimation accuracy and reliability based on estimation algorithms [150]. The effectiveness of vehicle state estimation is expected to be further improved, providing more timely and accurate information input for the active safety control system of vehicles for intelligent driving.

Vehicle state estimation technology has been widely applied in active safety systems such as electronic stability control (ESC). In actual vehicle integration, signals from wheel speed sensors, steering angle sensors, yaw rate sensors, etc. are obtained through the vehicle CAN bus. A state observer based on the vehicle dynamics model is used to estimate the actual driving state of the vehicle in real time, such as side slip angle, yaw rate, etc., and compare it with the ideal state. When an unstable state is detected in the vehicle, the ESC system can adjust the braking torque of each wheel in a timely manner to restore the stability of the vehicle. To meet the performance requirements under complex road conditions, some vehicles adopt adaptive control algorithms that can adjust control parameters in real time according to different road adhesion coefficients and vehicle load changes, optimizing the active safety performance of the vehicle. For example, Bosch’s ninth generation ESP system has improved the recognition accuracy and control effect of vehicle status through continuous algorithm optimization, effectively reducing the risk of vehicle loss of control in various driving conditions.

Vehicle state estimation technology still faces many challenges in practical integration and application processes. First, the noise and errors in sensor data in actual vehicle systems have a significant impact on the accuracy of state estimation. For example, GPS signals may be interfered with or lost in environments such as urban canyons and tunnels, which can affect the accuracy of vehicle location estimation. Second, the dynamic characteristics of vehicles under complex road conditions increase the difficulty of state estimation. For example, on slippery road surfaces or in emergency obstacle avoidance situations, the tire road adhesion coefficient of the vehicle may change, and traditional fixed model-based state estimation algorithms may not be able to accurately track the vehicle’s state. Furthermore, the real-time requirement of the vehicle state estimation system is also a challenge. In high-speed driving and complex driving scenarios, state estimation algorithms need to process a large amount of sensor data and output accurate estimation results in a very short time, which puts high demands on the computational efficiency and hardware performance of the algorithm.

To address the above challenges, researchers have proposed various solutions. In terms of data processing, adopting multi-sensor fusion technology can effectively reduce the impact of single sensor noise and error on state estimation. By designing sensor fusion algorithms reasonably, such as Kalman filtering and its improved algorithms, data level fusion, and decision level fusion, the advantages of each sensor can be fully utilized to improve the reliability and accuracy of state estimation. In terms of modeling, building more refined and adaptive vehicle dynamics models should be considered. For example, introducing machine learning methods for online identification and updating of vehicle dynamics models is important to better adapt to changes in vehicle dynamic characteristics under different road conditions and driving conditions. Meanwhile, the structure and parameters of the estimation algorithm should be optimized to improve its computational efficiency and convergence speed in order to meet the real-time requirements in practical applications. In addition, hardware optimization and redundancy design are also key measures to ensure the reliability of vehicle state estimation systems. The use of high-performance computing chips and low latency sensors, as well as the design of sensor redundancy backup mechanisms, can to some extent improve the system’s anti-interference and fault tolerance capabilities.

### 5.3. Applications for Vehicle Condition Monitoring and Fault-Tolerant Control

Vehicle state estimation technology is of great significance in modern vehicle fault diagnosis, state monitoring, and fault-tolerant control [43,46,47,48,54]. Through continuous and accurate monitoring and analysis, a real-time grasp of vehicle operation status and fault information can be obtained. The various sensors installed on the vehicle can monitor various parameters of the vehicle in real time, providing a real-time and accurate data foundation for vehicle state estimation, helping to detect potential faults in a timely manner and make corresponding decision-making judgments. In recent years, vehicle state diagnosis models have been constructed by applying machine learning techniques. By learning the data characteristics of vehicles under normal operation and fault conditions, the occurrence of faults can be automatically identified and determined [58,63,64,68,69,70,71]. Machine learning algorithms such as the threshold method, time series analysis, and deep learning, are widely used in fault detection, effectively improving the accuracy and efficiency of fault recognition.

Meanwhile, vehicle state estimation technology can also shine in the field of predictive maintenance. Through real-time monitoring and in-depth analysis of vehicle status, this technology can predict the occurrence of potential faults and provide forward-looking guidance for vehicle maintenance [126,127,159,160,161]. This preventive maintenance strategy effectively reduces the vehicle failure rate, improves the overall efficiency and safety of vehicle use, and greatly extends the service life of the vehicle. Hongao Liu et al. [127] proposed a battery health status estimation method based on a data-driven and deep fusion transfer learning network algorithm that achieved accurate monitoring of battery status.

The combination of vehicle state estimation technology and fault-tolerant control technology is also an important application scenario. The advantages of information acquisition and fault tolerance mechanisms should be fully integrated and utilized to improve the safety and reliability of vehicles. Among them, state estimation technology provides accurate vehicle state information for fault-tolerant control, enabling fault-tolerant control to achieve more accurate fault diagnosis and obtain reliable fault information [162,163,164]. Fault-tolerant control technology can reduce the impact of faults on vehicle operation and ensure vehicle safety and stability by adjusting control strategies based on accurate vehicle status and fault information when faults occur. Te Chen et al. [47] proposed a passive fault-tolerant control method for steering system actuators based on the vehicle center of mass sideslip angle and steering system fault estimation. The estimator based on Kalman filtering provides the required key driving states and fault estimates for the vehicle control system. Shenguang He et al. [18] proposed a fault detection and fault-tolerant control strategy for intelligent vehicle steering system faults that is based on bidirectional long short-term memory and sequence probability ratio testing algorithms to achieve fault estimation and diagnosis.

Real-time monitoring of key components and systems in actual vehicles is achieved through the integration of on-board diagnostic systems (OBDs) and vehicle state estimation technology. For example, by utilizing sensor data from the engine management system and combining it with state estimation algorithms, the combustion state and output power of the engine can be estimated to detect potential faults in advance. In terms of fault-tolerant control, when key sensors or actuators of the vehicle fail, fault-tolerant control strategies based on state estimation can utilize redundant information and estimated values to maintain the basic driving functions of the vehicle and ensure driving safety. For example, when the angle sensor of the steering system malfunctions, the auxiliary steering controller uses the estimated values of the vehicle’s side slip angle and yaw rate provided by the vehicle state estimation module to achieve steering control under the fault and reduce the impact of the fault on the vehicle’s driving. At the same time, vehicle manufacturers establish remote monitoring platforms to collect real-time vehicle status monitoring data, conduct big data analysis, and provide fault warnings, promptly notify vehicle owners for maintenance. This also improves the reliability and service life of vehicles.

## 6. The Challenges and Future Trends of Vehicle State Estimation Technology

This article provides a detailed summary and analysis of the research status of vehicle state estimation technology from the perspectives of estimation objects, vehicle characteristics, and estimation algorithms and discusses the main application scenarios of vehicle state estimation technology at present. The current challenges and promising directions for the development of vehicle state estimation technology are summarized as follows.

### 6.1. Research Challenges

#### 6.1.1. The Fusion Processing Method of Multimodal Data

Faced with increasingly redundant multimodal data, there are many urgent problems that need to be solved. On the one hand, there are differences in data collection format, frequency, and accuracy, and implementation consistency is difficult to guarantee. At the same time, sensor signals also have uncertainty. On the other hand, multimodal data contain complex dynamic changes, which pose significant challenges to data fusion processing and optimization. Currently, improving the robustness and adaptability of data fusion algorithms to meet the needs of different scenarios and complex environments, as well as enhancing the representational power of data-driven learning methods and reducing computational complexity, are all issues that require in-depth research and exploration.

#### 6.1.2. The Vehicle State Estimation Ability in Complex Environments

Under complex working conditions, such as high speed and severe steering, the vehicle model exhibits strong nonlinearity and coupling of dynamic parameters, which greatly reduces the accuracy of model-based observer estimation. Moreover, in large-scale complex road conditions such as rain, snow, and slippery conditions, a single machine vehicle state observer is difficult to apply to all complex scenarios, and the dynamic estimation effect is unsatisfactory. This seriously affects the effectiveness of vehicle state estimation in complex environments and is currently an urgent technical challenge that needs to be overcome.

#### 6.1.3. The Combination of Vehicle State Estimation Technology and the Trend of Vehicle Electrification and Intelligence

With the development of vehicle electrification and intelligence, the comprehensive utilization of various sensor data in state estimation can obtain comprehensive and accurate vehicle dynamic information. However, how to integrate multi-source sensor information while balancing accuracy, reliability, and real-time performance has become a key challenge. In addition, the introduction of deep learning and reinforcement learning techniques brings opportunities, but also presents new challenges for vehicle state estimation. Especially considering factors such as high latitude, strong nonlinearity, and strong dynamic characteristics of vehicle model parameters, improving the high representation ability, optimization accuracy, and speed of data-driven algorithms has become an urgent problem to be solved.

### 6.2. Future Trends

#### 6.2.1. The Fusion Processing Method of Multimodal Data

Future research directions can focus on developing more efficient and robust data-driven algorithms to achieve the fusion and optimization of multimodal data. At the same time, exploring lightweight deep learning models or optimization methods to reduce computational complexity and improve real-time performance is expected to make breakthroughs in this field, promoting the development of vehicle state estimation technology in multimodal data processing.

#### 6.2.2. Vehicle State Estimation Abilities in Complex Environments

Given the challenges posed by complex environments, future research will focus on innovating refined modeling methods for vehicle dynamics, exploring more accurate and effective ways to construct vehicle model mechanisms and optimize model parameters, thereby improving the accuracy and stability of vehicle state estimation. In addition, by combining the basic model or pre-trained dynamic model, the performance of the driving model and data fusion estimation algorithm can be further improved. At the same time, closed-loop fusion estimation strategies suitable for various working conditions can be designed based on actual scenarios, and the adaptability of the estimation system can be improved through information redundancy, thereby enhancing the ability of vehicle state estimation in complex environments.

#### 6.2.3. The Combination of Vehicle State Estimation Technology and the Trend of Vehicle Electrification and Intelligence

In the wave of vehicle electrification and intelligence, the future will further explore how to better integrate multi-source sensor information, balance accuracy, reliability, and real-time performance. Integration with digital twin platforms can provide virtual environment support for data fusion optimization, while edge-deployable lightweight state estimators can ensure real-time and efficient processing of multimodal data. At the same time, with the continuous development of deep learning and reinforcement learning technologies, using large-scale data training to accurately characterize the parameter mapping relationship of vehicles under different road conditions and states and constructing dynamic relationship networks for various state parameters of vehicles is expected to become an important development direction, helping the deep integration and collaborative development of vehicle state estimation technology with the trends of vehicle electrification and intelligence.

## 7. Conclusions

As one of the core technologies in the field of modern vehicle engineering, the importance of vehicle state estimation technology is increasingly prominent. This article comprehensively discusses the research progress of vehicle state estimation technology from three perspectives: estimation objects, vehicle characteristics, and estimation algorithms. At the same time, combined with current technological hotspots and industry development trends, it analyzes the main application scenarios, current technical challenges, and future development trends of vehicle state estimation technology. The summary content of this article can help professionals in the industry to grasp the overall technological trends in this field and inspire new ideas and insights for their subsequent research.

(1). From the perspective of the research object of vehicle state estimation, the current research content includes vehicle attitude, key dynamic parameters of chassis components related to vehicle motion and stability, as well as driving environment parameters in intelligent driving scenarios.
(2). From the perspective of vehicle characteristics, existing research mainly focuses on vehicle dynamics coupling characteristics, vehicle multi-source information redundancy characteristics, and vehicle state transition characteristics.
(3). From the perspective of estimation algorithms, currently there are mainly model-based Kalman filtering algorithms and nonlinear observation algorithms, data-driven-based machine learning algorithms, and fusion estimation algorithms driven by both mechanism models and data.
(4). The main value of state estimation technology is to replace high cost sensors with state estimation techniques or to improve the accuracy of obtaining key parameters and vehicle states using redundant data and optimization algorithms. At present, it has been fully integrated and widely applied in vehicle autonomous driving scenarios, active safety control systems, as well as vehicle status monitoring and fault-tolerant control.
(5). With the development trend of vehicle electrification, intelligence, and networking, the objects and application scenarios of vehicle state estimation are expected to be further expanded, and the dynamic coupling of parameters and multi-source data characteristics will become more complex. At this point, the optimization estimation algorithm under the dual driven concept of model and data will be an important research direction for achieving technological breakthroughs.

## Figures and Tables

**Table 2:** Overview of vehicle state estimation methods from different perspectives.

Perspective	Method	References
Estimation means	Based on kinematic models	[4,6,11,15,23,24,25,26,30,32,33,50]
	Based on dynamic models	[1,2,5,8,9,10,13,27,28,29,31,34,35,36,37,38,39,46,55]
	Combining kinematic and dynamic models	[12,14,16,17,18,19,20,21,28,40,41,42,43,44,45]
Applied algorithm	Kalman filter and its improved form	[6,8,12,13,40,41,42,43,45,46,47,72,73,74,77,81,85,92,96,97,110,112,113,121,122,123,124,125]
	Robust estimation algorithm	[1,25,28,29,31,34,36,37,38,39,41,42,79,82,83,108,109,116,117]
	Sliding-mode algorithm, least square method, and other nonlinear estimation algorithms	[2,3,4,5,7,17,20,30,33,35,36,43,44,62,67,84,93,94,95,115,118,119,120]

**Table 3:** Comparison between different estimation algorithms.

Algorithm Category	Method	Disadvantages
EKF	High computational efficiency; good adaptability to weak nonlinear systems	Large linearization errors in strongly nonlinear systems; limited observability; average robustness to model noise
UKF/CKF	Good estimation accuracy and stability; strong adaptability to moderately nonlinear systems	High computational cost; possible divergence or accuracy reduction in high-dimensional state spaces
PF	Suitable for strongly nonlinear systems; good noise robustness	Prone to sample degradation in high-dimensional state spaces
Deep Learning	Can handle complex sensor data; strong nonlinear mapping capability	High computational cost; complex model training
Reinforcement Learning	Can adaptively adjust estimation strategies; suitable for dynamic environments	High requirements for data quality and annotation accuracy
Hybrid Optimization Algorithm	Fully utilizes the advantages of both mechanism- and data-driven methods; improves estimation accuracy and robustness	Performance depends on the quality of data preprocessing and algorithm selection

## Data Availability

The authors confirm that the data supporting the findings of this study are available within the paper.
